# Inflammatory markers for predicting overall survival in gastric cancer patients: A systematic review and meta-analysis

**DOI:** 10.1371/journal.pone.0236445

**Published:** 2020-07-27

**Authors:** Mi-Rae Kim, A-Sol Kim, Hye-In Choi, Jae-Hun Jung, Ji Yeon Park, Hae-Jin Ko

**Affiliations:** 1 Department of Family Medicine, Kyungpook National University Hospital, Daegu, Republic of Korea; 2 Department of Family Medicine, School of Medicine, Kyungpook National University, Daegu, Republic of Korea; 3 Department of Family Medicine, Kyungpook National University Chilgok Hospital, Daegu, Republic of Korea; 4 Department of Surgery, School of Medicine, Kyungpook National University, Daegu, Republic of Korea; 5 Gastric Cancer Center, Kyungpook National University Chilgok Hospital, Daegu, Republic of Korea; Faculty of Medical Science - State University of Campinas, BRAZIL

## Abstract

Systemic inflammatory biomarkers have begun to be used in clinical practice to predict prognosis and survival of cancer patients, but the approach remains controversial. We conducted a meta-analysis to determine the predictive value of the c-reactive protein (CRP), neutrophil-to-lymphocyte ratio (NLR), and Glasgow prognostic score (GPS)/modified Glasgow prognostic score (mGPS) in the clinical outcome of gastric cancer (GC) patients. We searched literature databases to identify relevant studies. All articles identified in the search were independently reviewed based on predetermined selection criteria. Meta-analysis was conducted to calculate the hazard ratio (HR) and 95% confidence intervals (CI) of overall survival of the included studies. A total of 41 eligible cohort studies, involving a total of 18,348 patients meeting the inclusion criteria, were considered for meta-analysis. Increases in CRP (HR = 1.654, 95% CI: 1.272–2.151), NLR (HR = 1.605, 95% CI: 1.449–1.779), and GPS/mGPS (HR = 1.648, 95% CI: 1.351–2.011) were significantly associated with poorer survival in patients with GC. Substantial heterogeneities were noted in all three markers (I^2^ = 86.479%, 50.799%, 69.774%, in CRP, NLR, and GPS/mGPS, respectively). Subgroup analysis revealed a significant positive correlation between each marker and poor survival, regardless of country, study quality, cancer stage, study design, or the inclusion of patients undergoing chemotherapy. This meta-analysis demonstrates that CRP, NLR, and GPS/mGPS are associated with poor survival in patients with GC. Further prospective studies using standardized measurements are warranted to conclude the prognostic value of various inflammatory markers.

## Introduction

The incidence of gastric cancer (GC) has declined in recent decades, and newer diagnostic methods with improved sensitivity and specificity have contributed to the early diagnosis and treatment of GC [1–3]. However, because GC is often diagnosed at an advanced stage, it remains a major health problem in many countries around the world [4]. It is the fifth most common cancer diagnosed every year, with about one million (5.7%) new cases globally, and according to WHO database, it was the third leading cause of cancer deaths (783,000 deaths, 8.2%) in 2018 [5]. Nearly one-third of GC patients undergo curative-intent surgery or neoadjuvant therapies, including systemic chemotherapy and radiotherapy, but treatment outcomes remain poor, largely due to distant metastasis or local tumor recurrence [6,7]. The 5-year survival rate of advanced or metastatic GC is about 5–20%, with median overall survival (OS) less than 1 year [7].

Tumor stage can be used to predict prognosis of GC and determine the optimal treatment strategy; however, prognosis differs even among patients with cancers of the same stage [8]. In addition, pathological tumor stage, metastatic lymph node count, and depth of tumor invasion, all of which have a significant impact on the prognosis of GC patients, can only be properly confirmed postoperatively. On the other hand, preoperative evaluation of TNM stage cannot accurately predict the postoperative survival rate and may lead to several biases [9]. Increasing amounts of research are addressing how tumor oncological features and host-response factors are involved in the relationship between cancer and inflammation [10]. Moreover, although the Eastern Cooperative Oncology Group performance status (ECOG‑ps) is a commonly used predictor of prognosis and treatment outcome for cancer patients, ECOG-ps does not often reflect weight loss; thus, performance status is subjectively assessed [11]. These issues limit the use of ECOG-ps as a prognostic factor.

Cancer is intimately associated with inflammation [12,13]. Accordingly, markers of systemic inflammation such as c‑reactive protein (CRP), albumin, neutrophils, lymphocytes, neutrophil-to-lymphocyte ratio (NLR), Glasgow prognostic score (GPS), modified Glasgow prognostic score (mGPS), platelet-to-lymphocyte ratio (PLR), and interleukin-6 (IL-6) have been studied as local and systemic indicators of the inflammatory response, with the ultimate goal of identifying new prognostic factors for cancers [14–16].

Among them, CRP, NLR, and GPS/mGPS are useful, inexpensive, and easily-accessible markers that have several advantages relative to currently widely used markers such as TNM staging and ECOG‑ps [17,18]. Hence, we sought to systematically review the literature on the effects of these three inflammatory markers in GC patients and to estimate the influence of the proposed markers as indicators of OS of GC.

## Materials and methods

### Search strategy and study selection

A systematic search of the literature for related research published since 1990 was conducted using PubMed, SCOPUS, and Google Scholar. The following words were used as keywords: “gastric cancer,” “gastric carcinoma,” “gastro esophageal cancer,” “gastrointestinal malignancies,” “CRP,” “c-reactive protein,” “NLR,”, “GPS,” “Glasgow prognostic score,” “inflammation-based factors,” “inflammatory markers,” “systemic inflammation,” “inflammatory parameters,” and “plasma levels of cytokines” (before January 2020, see S1 Appendix). In addition, the references in the articles we found were also reviewed to collect further related studies that were not included in the above database. Three authors independently searched the literature and no limitations were imposed on date or language (MRK, HJK, and JHJ). This meta-analysis was carried out in accordance with the statement of Preferred Reporting Items for Systematic Reviews and Meta-Analyses (PRISMA, see S2 Appendix) statement [19].

### Inclusion and exclusion criteria

The criteria for selecting articles for our analysis were as follows: (i) all patients must have been diagnosed with GC based on histopathological examination; (ii) studies must be prospective or retrospective cohort designs evaluating OS according to CRP, NLR, or GPS/mGPS; (iii) enough data must be provided to calculate the hazard ratio (HR) with 95% confidence intervals (CIs) reported, (iv) the full text article must be available. Titles and abstracts of the identified studies were evaluated by three independent investigators (MRK, HJK, and JHJ). Subsequently, the full texts of the included studies were retrieved for comprehensive evaluation. Any discrepancies between the three investigators were resolved by three of the other authors (HIC, ASK and JYP) to obtain a consensus.

Three authors (MRK, HJK, and JHJ) independently extracted eligible studies and collected the required information according to the following details: Studies based on the following criteria were excluded from the meta-analysis: (i) duplication; (ii) case reports, editorials, conference abstracts, or reviews; (iii) studies with insufficient data for estimating the HR with 95% CIs; (iv) articles written in languages other than English; and (v) nonhuman research or irrelevant studies (vi) studies including GC patients with inflammatory diseases such as infection, autoimmune disease, or acute myocardial infarction.

### Data extraction and quality assessment

Data were evaluated and extracted from each study. All studies were double-checked, and differences were resolved by discussion and consensus. For each study, the following information was recorded: publication details (including name of first author), year of publication, study design, characteristics of the studied population (including mean age and age range), country of origin, HR of each inflammation marker for OS (as well as their 95% CIs and P values), outcome, and cut-off value used to define high levels of inflammatory markers. If several estimates were presented in the same article, we selected the highest one (multivariate analysis was superior to univariate analysis).

The methodological quality of the studies was assessed using the Newcastle–Ottawa Scale (NOS) [20]. NOS ranges from 0 up to 9 stars. There are no definite criteria for determining a high-quality study in NOS. In this study, the mean of NOS was 7.3 stars in CRP and 7.5 stars in both NRS and GPS/mGPS. Based on this data, we decided the NOS cut-off for a high-quality study to be ≥8 stars for each marker.

### Statistical analysis

Meta-analysis was performed using a random-effects model with the DerSimonian–Laird method [21] to estimate the summary HR and 95% CI. Pooled HRs and corresponding 95% CIs were used to evaluate the relationship between level of inflammatory markers and prognosis of patients with GC. P < 0.05 was considered statistically significant. Heterogeneity between studies was assessed using the Cochran's Q-test and Higgins I-squared statistics to measure the extent of variation not due to chance alone.

In the presence of heterogeneity, subgroup analyses were performed to access the prognostic value of CRP, NLR, and GPS/mGPS based on characteristics such as county, study quality, cancer stage, study design, cut-off values, and the inclusion of patients with chemotherapy, as well as to assess the potential cause of variation in the study results. Publication bias was evaluated using funnel plots, Egger's test and Begg's test [22]. All statistical analyses were performed using CMA (Comprehensive Meta-Analysis) Version 2.2.064.

## Results

### Literature search and study selection

**Fig 1:**
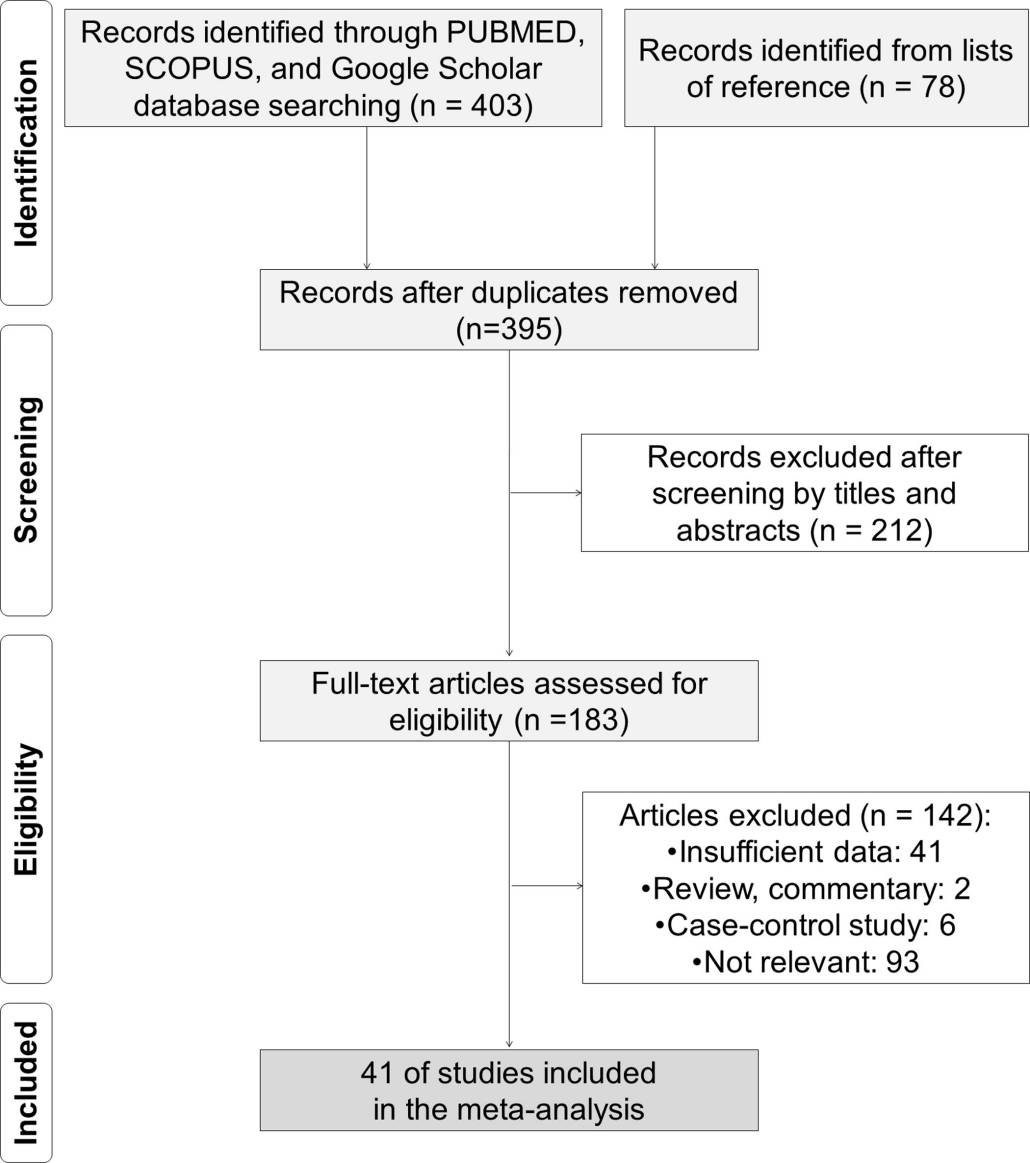
Flow diagram of the literature search and selection of studies for the meta-analysis.

The first 481 potentially relevant articles were identified through database searches and reference lists: PubMed, n = 232; SCOPUS, n = 104; Google scholar, n = 67; reference lists, n = 78. After removal of duplicates, 395 citations were identified through the systematic literature searches. Of these, 212 were excluded because they were considered irrelevant based on the content of their titles and abstracts, and of the remaining 183, 41 were excluded because they did not provide sufficient data for estimation of HR and 95% CI; eight were excluded for being reviews, invited commentary, or case-control studies; and 93 were judged to be irrelevant after reviewing the full text. Ultimately, we identified 41 full-text articles that met the inclusion criteria for our meta-analysis. A flow chart of the literature identification process is shown in Fig 1 (S3 Appendix).

The 41 studies, which involved a total of 18,348 patients, were published between 2007 and 2020 in six countries, including China (thirteen studies), South Korea (seven studies), Japan (16 studies), Italy (two studies), Germany (one study), and the UK (two studies). All studies provided an association between inflammation markers and OS in GC patients. Overall, the quality of evidence was sufficient to study the effects of CRP, NLR, and GPS/mGPS on the survival of GC patients. The cut-off value for CRP was < 1.0 mg/dL in five studies [23–27] and ≥ 1.0 mg/dL in six studies [28–33]. The cut-off value for NLR was <3 mg/dL in eleven studies [23,27,28,34–41], 3–4 mg/dL in nine studies [37,42–49], and ≥5 mg/dL in four studies [50–53]. The cut-off value for CRP of GPS/mGPS was <1.0 mg/dL in two studies [54,55] and ≥1.0 mg/dL in thirteen studies [7,28,39,43,50,52,53,56–61].

In all studies, NLR was defined as the absolute neutrophil count divided by the absolute lymphocyte count, based on pre-treatment laboratory data. GPS and mGPS were derived as previously described [11,62]. Patients with CRP elevation (> 1.0 mg/dL) and hypoalbuminemia (< 35 g/L) were assigned a GPS score of 2, whereas patients with only one of these biochemical abnormalities were assigned a GPS of 1. Patients without either of these abnormalities were assigned a score of 0. Patients with elevated CRP (> 1.0 mg/dL) were assigned an mGPS of 1 or 2 depending on the absence or presence of hypoalbuminemia (< 35 g/L), whereas patients with no CRP elevation (≤ 1.0 mg/dL) were assigned an mGPS of 0, even if hypoalbuminemia was present. HR and 95% CI were reported directly in all 41 studies. The clinical characteristics of the included studies are detailed in Table 1.

**Table 1:** Main characteristics of all eligible studies included in the meta-analysis.

Name	Study design	Country	Mean age (y)	Sample size (N)	CTx*	Stage^†^	Cut-off value		Quality assessment
**1. CRP**							CRP (mg/dL)		
Migita 2019^‡^ [33]	Retro	Japan	–	470	(−)	I	13.9		8
Guo 2018 [23]	Retro	China	–	1058	(−)	I-III	0.43		9
Kong 2016 [25]	Pro	China	60	72	(+)	IV	0.8		7
Sun 2016 [28]	Retro	China	59	873	(−)	I-III	1.0		7
Saito 2015 [32]	Retro	Japan	–	305	(−)	I-III	12.0		8
Ishizuka 2014 [24]	Retro	Japan	–	544	(−)	I-IV	0.3		7
Baba 2013 [29]	Retro	Japan	69	123	(−)	IV	1.7		7
Shimura 2012 [30]	Retro	Japan	–	61	(+)	IV	1.0		8
Iwasa 2011 [31]	Retro	Japan	58	79	(−)	IV	2.0		8
Fujitani 2011 [26]	Retro	Japan	62	53	(−)	IV	0.3		6
Mohri 2010 [27]	Retro	Japan	63.4	357	(−)	I-III	0.3		5
**2. NLR**							Ratio		
Miyamoto 2018 [47]	Retro	Japan	–	154	(−)	I-IV	3.50		7
Guo 2018 [23]	Retro	China	–	1058	(−)	I-III	2.50		9
Wen 2018 [52]	Retro	UK	66.1	723	(+)	I-IV	5.00		6
Choi 2017 [34]	Retro	South Korea	62	288	(−)	I-IV	2.70		6
Lieto 2017 [44]	Pro	Italy	–	401	(−)	I-IV	3.22		6
Liu 2017 [38]	Retro	China	58	1056	(−)	I-III	2.00		9
Mao 2017 [46]	Retro	China	59	337	(−)	I-IV	3.14		5
Liu 2016 [45]	Retro	China	57.7	817	(−)	I-III	3.71^⁋^		8
Sun 2016 [28]	Retro	China	59	873	(−)	I-III	2.30		7
Liu 2015 [39]	Retro	China	59	455	(−)	I-III	2.30		9
Qu 2015 [40]	Retro	China	59	1123	(−)	I-III	1.86		9
Aurello 2014 [53]	Retro	Italy	69	102	(−)	I-IV	5.00		9
Cho 2014 [42]	Retro	South Korea	55.44	268	(−)	IV	3.00		9
Jiang 2014 [35]	Pro	China	64	377	(−)	I-III	1.44		8
Mohri 2014 [48]	Retro	Japan	66	123	(−)	IV	3.10		7
Lee DY 2013 [63]	Retro	South Korea	57	220	(−)	I-IV	2.15		6
Lee S 2013 [37]	Pro	South Korea	55	174	(+)	I-IV	3.00		9
Dutta 2012 [50]	Pro	UK	–	120	(+)	I-III	5.00^⁋^		7
Jeong 2012 [43]	Retro	South Korea	52.5	104	(+)	IV	3.00		7
Wang 2012 [51]	Retro	China	–	324	(−)	III	5.00		7
Jung 2011 [36]	Retro	South Korea	63	293	(−)	III-IV	2.00		9
Mohri 2010 [27]	Retro	Japan	63.4	357	(−)	I-III	2.20		5
Shimada 2010 [49]	Pro	Japan	65	1028	(−)	I-IV	4.00		8
Yamanaka 2007 [41]	Pro	Japan	–	1220	(−)	IV	2.50		8
**3. GPS/mGPS**							CRP (mg/dL)	Albumin (g/L)	
Yuan 2018 [56]	Pro	China	–	384	(−)	IV	1.0	35	8
Powell 2018 [7]	Pro	UK	69	331	(−)	I-III	1.0	35	7
Wen 2018 [52]	Retro	UK	66.1	723	(+)	I-IV	1.0	35	6
Melling 2016 [57]	Pro	Germany	63.5	88	(−)	I-IV	1.0	35	8
Sun 2016 [28]	Retro	China	59	873	(−)	I-III	1.0	35	7
Liu 2015 [39]	Retro	China	59	455	(−)	I-III	1.0	35	9
Aurello 2014 [53]	Retro	Italy	69	102	(−)	I-IV	1.0	35	9
Hirashima 2014 [55]	Retro	Japan	68	294	(+)	I-IV	0.5	38	7
Li 2014 [58]	Pro	China	–	384	(−)	IV	1.0	35	9
Mimatsu 2014 [54]	Retro	Japan	–	36	(+)	IV	0.5	35	7
Dutta 2012 [50]	Pro	UK	–	120	(+)	I-III	1.0	35	7
Jeong 2012 [43]	Retro	South Korea	52.5	104	(+)	IV	1.0	35	7
Jiang 2012 [59]	Retro	China	–	1710	(−)	I-IV	1.0	35	6
Kubota 2012 [61]	Pro	Japan	62.9	1017	(−)	I-III	1.0	53	9
Hwang 2011 [60]	Pro	South Korea	59	402	(+)	IV	1.0	35	8

*(+), the inclusion of patients with neo- or adjuvant chemotherapy; (*–*), the exclusion of patients with chemotherapy or unspecificied.

^†^Stage IV includes recurrent, metastatic, primary unresectable, or incurable advanced gastric cancer.

^‡^Data of POD3 (post operation day 3) was used. ^⁋^Data of higher cut-off value was used. CRP, c-reactive protein; GPS, Glasgow prognostic score; mGPS, modified Glasgow prognostic score; NLR, neutrophil-to-lymphocyte ratio; Pro, prospective cohort study; Retro, retrospective cohort study.

### The prognostic value of CRP, NLR, and GPS/mGPS

**Fig 2:**
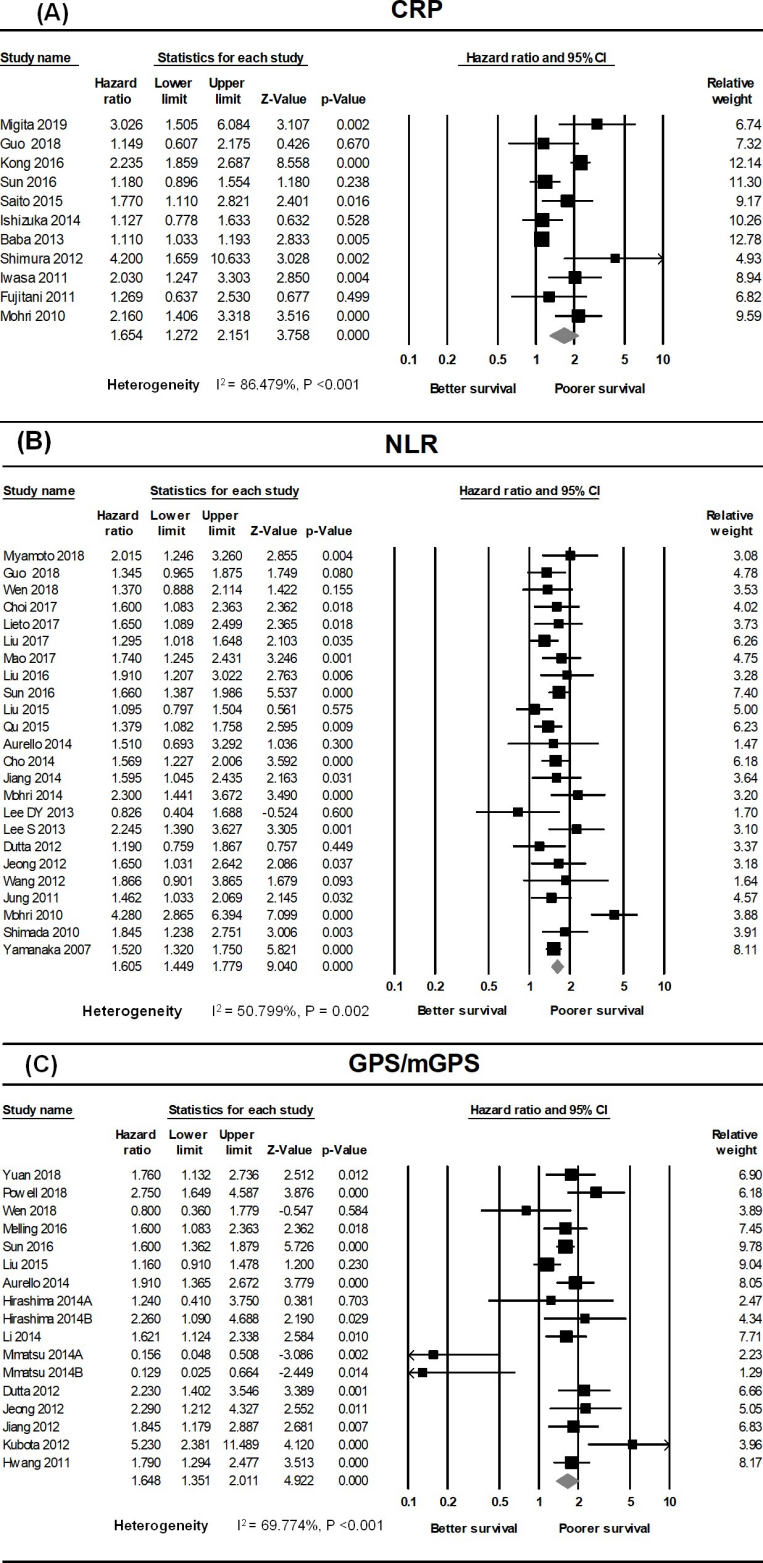
Forest plots showing results of studies on the association between elevated systemic inflammatory markers (CRP, NLR, GPS/mGPS) and overall survival in GC. Each study is indicated by a point estimate of the hazard ratio (HR) (the size of the square is proportional to the weight of each study) with 95% confidence intervals (CIs). (A) Forest plot of studies evaluating the association between elevated CRP and overall survival (OS) in patients with GC receiving various treatments. (B) Forest plot of studies evaluating the association between the neutrophil-to-lymphocyte ratio (NLR) and OS in GC. (C) Forest plot showing the prognostic effect of GPS/mGPS on the OS of patients with GC.

High CRP, NLR, and GPS/mGPS were positively correlated with poor OS in GC patients (Fig 2). Eleven cohort studies were used to investigate the association between CRP and OS in GC patients [23–33], which had substantial heterogeneity (I^2^ = 86.479%, P <0.001). The pooled analysis revealed that the HR was significantly higher in the elevated CRP group of GC patients than in the normal CRP group (HR = 1.654, 95% CI: 1.272–2.151, P <0.001) (Fig 2A).

A total of 24 studies provided sufficient data to assess the correlation between NLR and OS in GC patients [23,27,28,34–49,51–53,63,64]; we detected moderate heterogeneity between studies on the classified NLR (I^2^ = 50.799%, P = 0.002). The combined HR was markedly higher in GC patients with elevated NLR than in patients with normal NLR (HR = 1.605, 95% CI: 1.449–1.779, P <0.001) (Fig 2B).

Data suitable for investigating the correlation between GPS/mGPS and OS in GC patients were obtained from 15 studies [24,26–28,31,35–37,41,42,44,49,50,58,59], which had moderate heterogeneity (I^2^ = 69.774%, P <0.001). The pooled analysis revealed that the HR of patients with a GPS/mGPS score of 2 was 1.648 relative to patients with a GPS/mGPS score of 0 or 1 (HR = 1.648, 95% CI: 1.351–2.011, P <0.0001) (Fig 2C).

### Subgroup analysis

We next analyzed subgroup information related to several other relevant clinical features in the included studies; no significant changes were identified after stratification (Table 2). The subgroup analysis revealed that lower OS was associated with higher CRP, NLR, and GPS/mGPS in both low- and high-quality studies, and in both Asian and Western cohorts. Additionally, elevated CRP, NLR, and GPS/mGPS were significant negative predictors of prognosis at various stages of GC. Collectively, these inflammatory markers had prognostic value for GC outcomes regardless of country, quality of study, cancer stage, study design, or the inclusion of patients with neo- or adjuvant chemotherapy. The cut-off values used in the studies varied (Table 1). To evaluate the effect of cut-off values of proposed markers on prognosis, we also performed subgroup analysis based on the cut-off values. However, a subgroup analysis of the cut-off value of GPS/mGPS could not be performed because the criteria used were almost the same in all studies. Subgroup analysis based on the cut-off value also showed unchanged result that lower OS was related to elevated CRP and NLR.

**Table 2:** Subgroup analysis of meta-analysis.

Subgroup	Number of studies	Estimated effect size				Heterogeneity
		HR	95% CI	Z-value	P-value	I^2^ (%)
**1. CRP**						
**Quality of study**						
High	5	2.042	1.425–2.928	3.887	<0.001	42.515
Low	6	1.446	1.044–2.004	2.217	0.027	90.921
**Stage**						
Advanced*	6	1.649	1.112–2.445	2.489	0.013	91.546
Stage I–III	5	1.667	1.183–2.348	2.923	0.003	62.656
**Cut-off value (mg/dL)**						
CRP < 1.0	5	1.591	1.121–2.258	2.600	0.009	72.654
CRP ≥ 1.0	6	1.686	1.218–2.335	3.145	0.002	79.409
**Chemotherapy**^†^						
Included	2	2.582	1.536–4.341	3.579	<0.001	41.339
Excluded	9	1.457	1.174–1.809	3.413	0.001	68.040
**Study design**						
Prospective	1	2.235	1.859–2.687	8.558	<0.001	<0.001
Retrospective	10	1.545	1.226–1.948	3.684	<0.001	72.134
**2. NLR**						
**Quality of study**						
High	8	1.401	1.251–1.570	5.836	<0.001	5.709
Low	16	1.732	1.509–1.987	7.815	<0.001	54.168
**Stage**						
Advanced*	14	1.603	1.468–1.750	10.518	<0.001	<0.001
Stage I–III	10	1.586	1.288–1.953	4.348	<0.001	74.883
**Country**						
Asia	20	1.638	1.460–1.837	8.428	<0.001	57.571
Western	4	1.413	1.114–1.792	2.849	0.004	<0.001
**Cut-off value (mg/dL)**						
NLR ≤ 3	14	1.562	1.359–1.795	6.284	<0.001	66.679
NLR > 3	10	1.710	1.481–1.975	7.320	<0.001	<0.001
**Chemotherapy**^†^						
Included	4	1.550	1.190–2.019	3.252	<0.001	24.871
Excluded	20	1.615	1.443–1.809	8.310	<0.001	55.537
**Study design**						
Prospective	6	1.569	1.400–1.758	7.759	<0.001	<0.001
Retrospective	18	1.608	1.405–1.840	6.891	<0.001	59.778
**3. GPS/mGPS**						
**Quality of study**						
High	7	1.731	1.363–2.197	4.506	<0.001	65.037
Low	8	1.433	1.000–2.053	1.960	0.050	74.725
**Stage**						
Advanced*	10	1.474	1.132–1.918	2.884	0.004	64.636
Stage I–III	5	1.998	1.393–2.865	3.764	<0.001	81.592
**Country**						
Asia	10	1.559	1.205–2.018	3.375	<0.001	74.134
Western	5	1.851	1.387–2.470	4.184	<0.001	48.054
**Chemotherapy**^†^						
Included	6	1.149	0.679–1.943	0.517	0.605	77.853
Excluded	9	1.763	1.455–2.136	5.786	<0.0001	62.488
**Study design**						
Prospective	7	2.000	1.606–2.491	6.188	<0.001	42.906
Retrospective	8	1.319	0.976–1.784	1.800	0.072	74.911

*the inclusion of patients with stage IV gastric cancer.

^**†**^the inclusion of patients with neo- or adjuvant chemotherapy. CRP, c-reactive protein; GPS, Glasgow prognostic score; HR, hazard ratio; mGPS, modified Glasgow prognostic score; NLR, neutrophil-to-lymphocyte ratio.

### Publication bias

**Fig 3:**
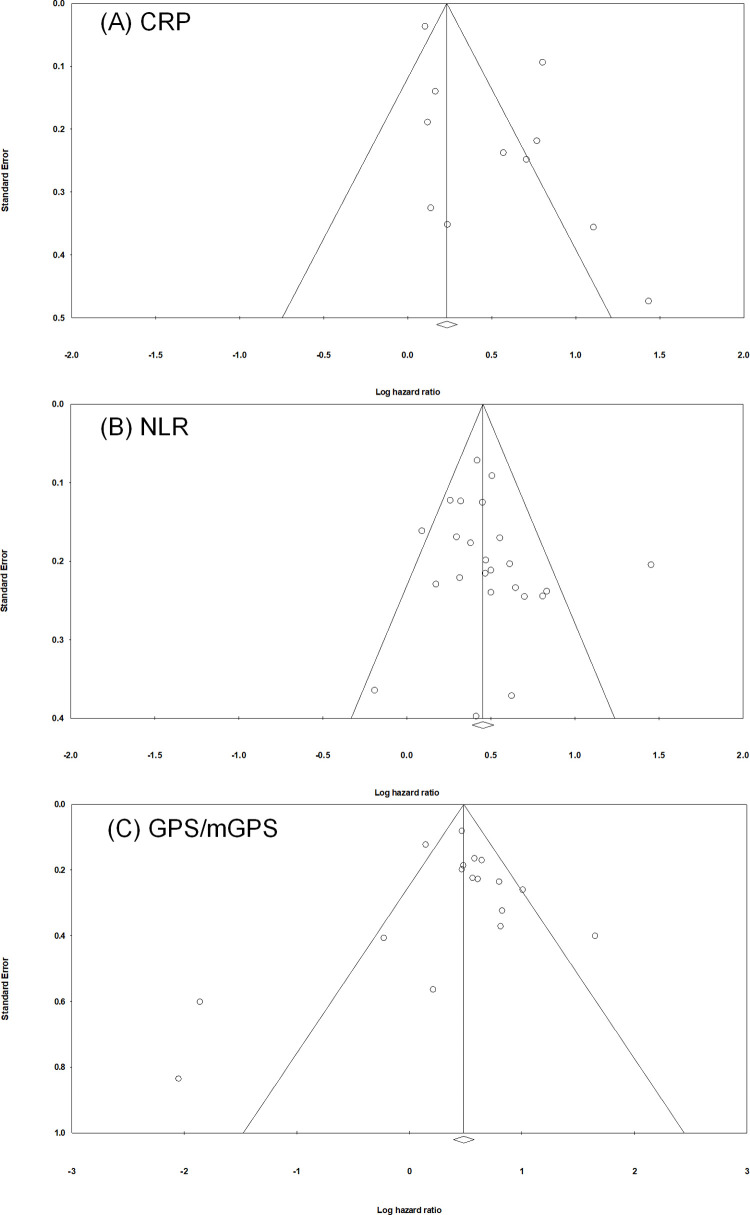
Funnel plots for assessing publication bias for meta-analysis of the correlation between OS and systemic inflammatory markers. (A) CRP, (B) NLR, and (C) GPS/mGPS. Each study is represented by one circle. The vertical line represents the pooled effect estimate.

The figure of the funnel plot was approximately symmetrical, and there was no evidence of obvious asymmetry. Begg’s test and Egger’s test revealed nonsignificant publication bias for each inflammatory marker (Begg's test: P = 0.213, 0.143, and 0.902 for CRP, NLR, and GPS/mGPS, respectively; Egger's test: P = 0.056, 0.395, and 0.731 for the CRP, NLR, and GPS/mGPS, respectively). Thus, our inspection of the funnel plots did not reveal any evidence of publication bias in this meta-analysis (Fig 3).

## Discussions

This meta-analysis, which included studies involving 18,348 participants, assessed the prognostic value of CRP, NLR, and GPS/mGPS in GC patients. We also performed subgroup analysis to assess the relationship between systemic inflammatory markers and OS. Higher levels of the three markers were associated with poor OS in GC. This finding suggests that CRP, NLR and GPS/mGPS could help in decision-making by assisting physicians in estimating GC status before and after surgery and chemotherapy.

The association of inflammation with cancer, which was deduced from the detection of infiltrating leukocytes in neoplastic tissues, was first reported by Rudolf Virchow in 1863 [65]. The cancer-related inflammatory response is a response to chemotherapy, as well as a non-specific response to promotion of cell proliferation, cell survival, epithelial–mesenchymal transition (EMT), tumor hypoxia, angiogenesis, tumor cell migration, invasion, activation of anti-apoptotic signaling pathways, and metastasis [66]. Cancer causes inflammation, which results in the activation of transcription factors that further increase the inflammatory response, such as nuclear factor-kB (NF-kB), signal transducer and activator of transcription 3 (STAT3), and hypoxia-inducible factor 1a (HIF1a) [67]. These transcription factors were modulated to produce several important tumor growth-promoting cytokines, including TNF-a, IL- 1β, and IL-6 [68]. Therefore, the complex and diverse neuroendocrinology and hematopoietic changes that occur during inflammation play important roles in both attenuating the immune response and increasing tumor growth [35].

Recently, CRP, NLR, and GPS/mGPS have attracted attention as prognostic factors in cancer. CRP, a sensitive and widely used systemic inflammatory marker, is mainly produced in the liver along with other acute-phase proteins synthesized by the liver in response to cytokines, including interleukin-1(IL-1), IL-6, and tumor necrosis factor-α (TNF-α) [69].

NLR can be used to symbolize the balance between activation of pro-tumor inflammatory pathways and antitumor immune function. Lymphopenia is an impaired cell-mediated immune response, whereas neutrophilia is a systematic inflammatory response. Neutrophilia can be induced by tumor-associated granulocyte colony stimulating factor (GCSF), which specifically acts on bone marrow granulocytic cells [70]. At the same time, neutrophilia can stimulate tumorigenesis by stimulating the secretion of vascular endothelial growth factor (VEGF). In addition, neutrophil-derived reactive oxygen species further decrease the adhesion-promoting properties of the extracellular matrix and inhibit tumor cell apoptosis through activation of NF-kB and STAT3 [71]. Lymphocytopenia caused by the systemic inflammatory response reflects significant decreases in the abundance of T4 helper lymphocytes and innate cellular immunity, manifested as an increase in the abundance of T8 suppressor lymphocytes [72]. Indeed, some studies have shown that an increase in neutrophil abundance suppresses the cytolytic activity of lymphocytes, natural killer cells, and activated T cells.

There is consistent evidence that hypo-albuminemia, a consequence of the systemic inflammatory response, is associated with gradual malnutrition and immune dysfunction in cancer patients and subsequent poor outcome, regardless of tumor stage [41]. Patients in a pre-cachectic state may undergo combination therapy that can delay the onset of cachexia and death. In addition to helping tumor treatment, GPS/mGPS can also be easily used to improve weight loss and poor performance status in patients with GC.

TNM staging and ECOG‑ps, which are primarily focused on the biological behavior and expression of the tumor itself, serve as bases for segmenting GC patients and determining appropriate therapies. This is an imperfect approach, however, because patients at the same stage can have different clinical outcomes [8]. Additionally, because biomarkers such as CA19-9, CEA, and AFP are generally expensive, they are not tested in routine pathological evaluation of GC. Thus, the introduction of a cost-effective and accessible laboratory index as an adjunct to the current tumor staging system is important for risk level assessments of GC patients.

Our analysis has several limitations, in particular because we focused on observational cohort studies that are more vulnerable to certain objections. First, in studies that did not specify in detail how they eliminated or reduced the rate of false positives, some tumors may have been misclassified by histology or location. Second, the cut-off values for defining high CRP, NLR, and GPS/mGPS were not uniform. However, the prognostic value of these markers was not affected because the majority of subgroup analyses did not yield a different outcome, indicating that the results were relatively conclusive. Third, this analysis included only those published in English, and small studies with cumulative results tend not to be published, leading to potential bias. However, we detected no significant publication bias related to any of the three markers, implying that this limitation is not significant. Fourth, in two of the studies [28,50], because CRP, NLR, and GPS/mGPS were not statistically significant in multivariate analysis, the statistical significance of these markers was estimated using univariate analysis. This may have impaired the accuracy of the pooled data. Even though we used the random-effects model for all meta-analysis and performed subgroup analysis with various factors [73], significant heterogeneity between studies is another potential limitation. Most of the included studies used the data measured before specific procedures, however, the time of data collection and follow-up period varied between studies. As the incidence of GC is particularly high in Asians [74], furthermore, most of the included studies in this meta-analysis were conducted in Asia (35 out of 41 studies). These factors including methodological diversities and racial differences might have influence on the heterogeneity. Finally, the study populations and patient selection criteria were not fully reported. Little information has been reported on the reproducibility of the tests, which can change the reliability of potential data. Moreover, because diagnostic tests may have different accuracies at distinctive stages of the disease, the results are affected by spectrum bias. Considering these limitations, further prospective studies with large subjects using standardized measurements are warranted to conclude prognostic value of the inflammatory markers.

Previous meta-analyses assessed some of these relationships and revealed that the systemic inflammatory markers had predictive value for OS of GC patients [75–77]. However, none of the three markers have been linked to the GC survival rate. This meta-analysis is the first to exclusively include three systemic inflammatory markers, making the results more powerful and robust. Although the number of GC patients we analyzed is small, we have synthesized previously published studies to produce more accurate and reliable results. Our results also reveal more predictive biomarkers that are easily accessible from peripheral blood samples, convenient, practical, inexpensive, precise, reproducible in clinical applications. We also evaluated prognosis using a new interpretation of cut-off values that are different from those reported previously.

In conclusion, our meta-analysis confirmed that elevations in CRP, NLR, and GPS/mGPS are associated with poorer survival outcome in GC patients, with a higher GPS/mGPS having a greater negative effect on overall survival. Subgroup analyses revealed that regardless of country, quality score, stage, or chemotherapy, higher levels of each inflammatory marker were associated with lower survival. Thus, interventions to modulate the inflammatory response and immune response before and after surgery could help to improve long-term cancer outcomes.

Although this study reached the conclusion that these inflammatory markers could serve as prognostic factors in patients with GC, potential confounding factors were not adequately taken into consideration. Therefore, we recommend that prospective studies and standardized surveys of GC be conducted in the future. Potential confounding factors such as age, sex, ethnicity, host factors, stage, adjuvant therapy, and effect modifiers should be examined. To provide conclusive information, future studies should also ensure an adequate sample size to take into account the frequency of inflammatory markers, the magnitude of the effect of interest, and the potential for interaction.

## Supporting information

S1 Appendix Search strategy. (DOCX)

S2 Appendix PRISMA checklist. (DOC)

S3 Appendix List of extracted studies. (DOCX)
