# Supplementary material for: Inflammatory markers for predicting overall survival in gastric cancer patients: A systematic review and meta-analysis
Source: PLoS One. 2020 Jul 27;15(7):e0236445. doi: 10.1371/journal.pone.0236445 (PMC7384660; doi:10.1371/journal.pone.0236445)
Supplement: S1 Appendix — (DOCX) [file pone.0236445.s001.docx]

**S1. Search Strategy**

The studies published until 20 January 2020 were searched.

**PubMed**

Search:

**("gastric cancer"[Title] OR "gastric carcinoma"[Title] OR "gastro esophageal cancer"[Title] OR "gastrointestinal malignancies"[Title]) AND ("crp"[Title] OR "c-reactive protein"[Tilte] OR "NLR"[Title] OR "GPS"[Title] OR "glasgow prognostic score"[Title] OR "inflammation-based factors"[Title] OR "inflammatory markers"[Tilte] OR "systemic inflammation"[Title] OR "inflammatory parameters"[Title])**

- 249 results

After applying only human (species), full text (text availability), medline (journal) and Enlglish (language) conditions.

- 163 results

**SCOPUS**

Search:

**(TITLE(gastric cancer) OR TITLE(gastric carcinoma) OR TITLE(gastro esophageal cancer)) AND (TITLE(crp) OR TITLE(c-reactive protein) OR TITLE(NLR) OR TITLE(GPS) OR TITLE(glasgow prognostic score) OR TITLE(inflammation-based factor) OR TITLE(inflammatory markers) OR TITLE(systemic inflammation) OR TITLE(inflammatory parameters))**

**-** 104 results

After applying only medicine (subject data), article (document data) and Enlglish (language) conditions

- 76 results

**Google Scholar**

As the search terms do not fit into the search box, the search is divided into two search terms for convenience.

Search:

1. allintitle: ("gastric cancer" OR "gastric carcinoma" OR "gastro esophageal cancer") AND ("CRP" OR "c-reactive protein" OR "NLR " OR "GPS " OR "glasgow score") –meta

- 59 results

2 allintitle: ("gastric cancer" OR "gastric carcinoma" OR "gastro esophageal cancer") AND ("glasgow score" OR "inflammation based factors "OR "inflammatory markers" OR "systemic inflammation" OR "inflammation parameters") -meta"

- 8 results
