# Supplementary material for: Inflammatory markers for predicting overall survival in gastric cancer patients: A systematic review and meta-analysis
Source: PLoS One. 2020 Jul 27;15(7):e0236445. doi: 10.1371/journal.pone.0236445 (PMC7384660; doi:10.1371/journal.pone.0236445)
Supplement: S3 Appendix — (DOCX) [file pone.0236445.s003.docx]

**S3. List of Extracted Studies**

- **C-reactive protein (CRP): 11 studies**

1. Migita K, Matsumoto S, Wakatsuki K, Kunishige T, Nakade H, Miyao S, et al. Postoperative Serum C-Reactive Protein Level Predicts Long-term Outcomes in Stage I Gastric Cancer. The Journal of surgical research. 2019;242:323-31.

2. Guo J, Chen S, Chen Y, Li S, Xu D. Combination of CRP and NLR: a better predictor of postoperative survival in patients with gastric cancer. Cancer Manag Res. 2018;10:315-21.

3. Kong F, Gao F, Chen J, Zheng R, Liu H, Li X, et al. Elevated serum C-reactive protein level predicts a poor prognosis for recurrent gastric cancer. Oncotarget. 2016;7(34):55765-70.

4. Sun X, Wang J, Liu J, Chen S, Liu X. Albumin concentrations plus neutrophil lymphocyte ratios for predicting overall survival after curative resection for gastric cancer. OncoTargets and therapy. 2016;9:4661-9.

5. Saito T, Kurokawa Y, Miyazaki Y, Makino T, Takahashi T, Yamasaki M, et al. Which is a more reliable indicator of survival after gastric cancer surgery: Postoperative complication occurrence or C-reactive protein elevation? Journal of surgical oncology. 2015;112(8):894-9.

6. Ishizuka M, Oyama Y, Abe A, Kubota K. Combination of platelet count and neutrophil to lymphocyte ratio is a useful predictor of postoperative survival in patients undergoing surgery for gastric cancer. Journal of surgical oncology. 2014;110(8):935-41.

7. Baba H, Kuwabara K, Ishiguro T, Hatano S, Matsuzawa T, Fukuchi M, et al. C-reactive protein as a significant prognostic factor for stage IV gastric cancer patients. Anticancer Research. 2013;33(12):5591-6.

8. Shimura T, Kitagawa M, Yamada T, Ebi M, Mizoshita T, Tanida S, et al. C-reactive protein is a potential prognostic factor for metastatic gastric cancer. Anticancer Research. 2012;32(2):491-6.

9. Iwasa S, Nakajima TE, Nakamura K, Takashima A, Kato K, Hamaguchi T, et al. Systemic chemotherapy for peritoneal disseminated gastric cancer with inadequate oral intake: a retrospective study. International journal of clinical oncology. 2011;16(1):57-62.

10. Fujitani K, Yamada M, Hirao M, Kurokawa Y, Tsujinaka T. Optimal indications of surgical palliation for incurable advanced gastric cancer presenting with malignant gastrointestinal obstruction. Gastric cancer : official journal of the International Gastric Cancer Association and the Japanese Gastric Cancer Association. 2011;14(4):353-9.

11. Mohri Y, Tanaka K, Ohi M, Yokoe T, Miki C, Kusunoki M. Prognostic significance of host- and tumor-related factors in patients with gastric cancer. World journal of surgery. 2010;34(2):285-90.

- **Neutrophil-to-lymphocyte ratio (NLR): 24 studies**

1. Miyamoto R, Inagawa S, Sano N, Tadano S, Adachi S, Yamamoto M. The neutrophil-to-lymphocyte ratio (NLR) predicts short-term and long-term outcomes in gastric cancer patients. European journal of surgical oncology : the journal of the European Society of Surgical Oncology and the British Association of Surgical Oncology. 2018;44(5):607-12.

2. Guo J, Chen S, Chen Y, Li S, Xu D. Combination of CRP and NLR: a better predictor of postoperative survival in patients with gastric cancer. Cancer Manag Res. 2018;10:315-21.

3. Wen J, Bedford M, Begum R, Mitchell H, Hodson J, Whiting J, et al. The value of inflammation based prognostic scores in patients undergoing surgical resection for oesophageal and gastric carcinoma. Journal of surgical oncology. 2018;117(8):1697-707.

4. Choi Y, Kim JW, Nam KH, Han SH, Kim JW, Ahn SH, et al. Systemic inflammation is associated with the density of immune cells in the tumor microenvironment of gastric cancer. Gastric cancer : official journal of the International Gastric Cancer Association and the Japanese Gastric Cancer Association. 2017;20(4):602-11.

5. Lieto E, Galizia G, Auricchio A, Cardella F, Mabilia A, Basile N, et al. Preoperative Neutrophil to Lymphocyte Ratio and Lymphocyte to Monocyte Ratio are Prognostic Factors in Gastric Cancers Undergoing Surgery. Journal of gastrointestinal surgery : official journal of the Society for Surgery of the Alimentary Tract. 2017;21(11):1764-74.

6. Liu X, Chen S, Liu J, Xu D, Li W, Zhan Y, et al. Impact of systemic inflammation on gastric cancer outcomes. PloS one. 2017;12(3):e0174085.

7. Mao M, Wei X, Sheng H, Chi P, Liu Y, Huang X, et al. C-reactive protein/albumin and neutrophil/lymphocyte ratios and their combination predict overall survival in patients with gastric cancer. Oncol Lett. 2017;14(6):7417-24.

8. Liu J, Geng Q, Chen S, Liu X, Kong P, Zhou Z, et al. Nomogram based on systemic inflammatory response markers predicting the survival of patients with resectable gastric cancer after D2 gastrectomy. Oncotarget. 2016;7(25):37556-65.

9. Sun X, Wang J, Liu J, Chen S, Liu X. Albumin concentrations plus neutrophil lymphocyte ratios for predicting overall survival after curative resection for gastric cancer. OncoTargets and therapy. 2016;9:4661-9.

10. Liu X, Sun X, Liu J, Kong P, Chen S, Zhan Y, et al. Preoperative C-Reactive Protein/Albumin Ratio Predicts Prognosis of Patients after Curative Resection for Gastric Cancer. Transl Oncol. 2015;8(4):339-45.

11. Qu JL, Qu XJ, Li Z, Zhang JD, Liu J, Teng YE, et al. Prognostic Model Based on Systemic Inflammatory Response and Clinicopathological Factors to Predict Outcome of Patients with Node-Negative Gastric Cancer. PloS one. 2015;10(6):e0128540.

12. Aurello P, Tierno SM, Berardi G, Tomassini F, Magistri P, D'Angelo F, et al. Value of preoperative inflammation-based prognostic scores in predicting overall survival and disease-free survival in patients with gastric cancer. Annals of surgical oncology. 2014;21(6):1998-2004.

13. Cho IR, Park JC, Park CH, Jo JH, Lee HJ, Kim S, et al. Pre-treatment neutrophil to lymphocyte ratio as a prognostic marker to predict chemotherapeutic response and survival outcomes in metastatic advanced gastric cancer. Gastric cancer : official journal of the International Gastric Cancer Association and the Japanese Gastric Cancer Association. 2014;17(4):703-10.

14. Jiang N, Deng JY, Liu Y, Ke B, Liu HG, Liang H. The role of preoperative neutrophil-lymphocyte and platelet-lymphocyte ratio in patients after radical resection for gastric cancer. Biomarkers : biochemical indicators of exposure, response, and susceptibility to chemicals. 2014;19(6):444-51.

15. Mohri Y, Tanaka K, Ohi M, Saigusa S, Yasuda H, Toiyama Y, et al. Identification of prognostic factors and surgical indications for metastatic gastric cancer. BMC cancer. 2014;14:409.

16. Lee DY, Hong SW, Chang YG, Lee WY, Lee B. Clinical significance of preoperative inflammatory parameters in gastric cancer patients. J Gastric Cancer. 2013;13(2):111-6.

17. Lee S, Oh SY, Kim SH, Lee JH, Kim MC, Kim KH, et al. Prognostic significance of neutrophil lymphocyte ratio and platelet lymphocyte ratio in advanced gastric cancer patients treated with FOLFOX chemotherapy. BMC cancer. 2013;13:350.

18. Dutta S, Crumley AB, Fullarton GM, Horgan PG, McMillan DC. Comparison of the prognostic value of tumour and patient related factors in patients undergoing potentially curative resection of gastric cancer. American journal of surgery. 2012;204(3):294-9.

19. Jeong JH, Lim SM, Yun JY, Rhee GW, Lim JY, Cho JY, et al. Comparison of two inflammation-based prognostic scores in patients with unresectable advanced gastric cancer. Oncology. 2012;83(5):292-9.

20. Wang DS, Ren C, Qiu MZ, Luo HY, Wang ZQ, Zhang DS, et al. Comparison of the prognostic value of various preoperative inflammation-based factors in patients with stage III gastric cancer. Tumour biology : the journal of the International Society for Oncodevelopmental Biology and Medicine. 2012;33(3):749-56.

21. Jung MR, Park YK, Jeong O, Seon JW, Ryu SY, Kim DY, et al. Elevated preoperative neutrophil to lymphocyte ratio predicts poor survival following resection in late stage gastric cancer. Journal of surgical oncology. 2011;104(5):504-10.

22. Mohri Y, Tanaka K, Ohi M, Yokoe T, Miki C, Kusunoki M. Prognostic significance of host- and tumor-related factors in patients with gastric cancer. World journal of surgery. 2010;34(2):285-90.

23. Shimada H, Takiguchi N, Kainuma O, Soda H, Ikeda A, Cho A, et al. High preoperative neutrophil-lymphocyte ratio predicts poor survival in patients with gastric cancer. Gastric cancer : official journal of the International Gastric Cancer Association and the Japanese Gastric Cancer Association. 2010;13(3):170-6.

24. Yamanaka T, Matsumoto S, Teramukai S, Ishiwata R, Nagai Y, Fukushima M. The baseline ratio of neutrophils to lymphocytes is associated with patient prognosis in advanced gastric cancer. Oncology. 2007;73(3-4):215-20.

- **Glasgow prognostic score (GPS)/modified Glasgow prognostic score (mGPS): 15 studies**

1. Yuan SQ, Nie RC, Chen YM, Qiu HB, Li XP, Chen XJ, et al. Glasgow Prognostic Score is superior to ECOG PS as a prognostic factor in patients with gastric cancer with peritoneal seeding. Oncol Lett. 2018;15(4):4193-200.

2. Powell A, Parkinson D, Patel N, Chan D, Christian A, Lewis WG. Prognostic Significance of Serum Inflammatory Markers in Gastric Cancer. Journal of gastrointestinal surgery : official journal of the Society for Surgery of the Alimentary Tract. 2018;22(4):595-605.

3. Wen J, Bedford M, Begum R, Mitchell H, Hodson J, Whiting J, et al. The value of inflammation based prognostic scores in patients undergoing surgical resection for oesophageal and gastric carcinoma. Journal of surgical oncology. 2018;117(8):1697-707.

4. Melling N, Gruning A, Tachezy M, Nentwich M, Reeh M, Uzunoglu FG, et al. Glasgow Prognostic Score may be a prognostic index for overall and perioperative survival in gastric cancer without perioperative treatment. Surgery. 2016;159(6):1548-56.

5. Sun X, Wang J, Liu J, Chen S, Liu X. Albumin concentrations plus neutrophil lymphocyte ratios for predicting overall survival after curative resection for gastric cancer. OncoTargets and therapy. 2016;9:4661-9.

6. Liu X, Sun X, Liu J, Kong P, Chen S, Zhan Y, et al. Preoperative C-Reactive Protein/Albumin Ratio Predicts Prognosis of Patients after Curative Resection for Gastric Cancer. Transl Oncol. 2015;8(4):339-45.

7. Aurello P, Tierno SM, Berardi G, Tomassini F, Magistri P, D'Angelo F, et al. Value of preoperative inflammation-based prognostic scores in predicting overall survival and disease-free survival in patients with gastric cancer. Annals of surgical oncology. 2014;21(6):1998-2004.

8. Hirashima K, Watanabe M, Shigaki H, Imamura Y, Ida S, Iwatsuki M, et al. Prognostic significance of the modified Glasgow prognostic score in elderly patients with gastric cancer. Journal of gastroenterology. 2014;49(6):1040-6.

9. Li QQ, Lu ZH, Yang L, Lu M, Zhang XT, Li J, et al. Neutrophil count and the inflammation-based glasgow prognostic score predict survival in patients with advanced gastric cancer receiving first-line chemotherapy. Asian Pacific journal of cancer prevention : APJCP. 2014;15(2):945-50.

10. Mimatsu K, Oida T, Fukino N, Kano H, Kawasaki A, Kida K, et al. Glasgow prognostic score is a useful predictive factor of outcome after palliative gastrectomy for stage IV gastric cancer. Anticancer Research. 2014;34(6):3131-6.

11. Dutta S, Crumley AB, Fullarton GM, Horgan PG, McMillan DC. Comparison of the prognostic value of tumour and patient related factors in patients undergoing potentially curative resection of gastric cancer. American journal of surgery. 2012;204(3):294-9.

12. Jeong JH, Lim SM, Yun JY, Rhee GW, Lim JY, Cho JY, et al. Comparison of two inflammation-based prognostic scores in patients with unresectable advanced gastric cancer. Oncology. 2012;83(5):292-9.

13. Jiang X, Hiki N, Nunobe S, Kumagai K, Kubota T, Aikou S, et al. Prognostic importance of the inflammation-based Glasgow prognostic score in patients with gastric cancer. British journal of cancer. 2012;107(2):275-9.

14. Kubota T, Hiki N, Nunobe S, Kumagai K, Aikou S, Watanabe R, et al. Significance of the inflammation-based Glasgow prognostic score for short- and long-term outcomes after curative resection of gastric cancer. Journal of gastrointestinal surgery : official journal of the Society for Surgery of the Alimentary Tract. 2012;16(11):2037-44.

15. Hwang JE, Kim HN, Kim DE, Choi HJ, Jung SH, Shim HJ, et al. Prognostic significance of a systemic inflammatory response in patients receiving first-line palliative chemotherapy for recurred or metastatic gastric cancer. BMC cancer. 2011;11:489.
